# The Effect of Initial Closed Reduction Quality on Patient Outcomes Following Ankle Fractures: A Retrospective Study

**DOI:** 10.7759/cureus.79418

**Published:** 2025-02-21

**Authors:** Andrea H Johnson, Jane C Brennan, Daniel Boudani, Adrienne A Spirt, David Keblish, Justin J Turcotte, Elizabeth Friedmann

**Affiliations:** 1 Department of Orthopedics, Anne Arundel Medical Center, Annapolis, USA; 2 Department of Orthopedic Research, Anne Arundel Medical Center, Annapolis, USA; 3 Department of Orthopedic Surgery, Anne Arundel Medical Center, Annapolis, USA; 4 Department of Orthopedic and Surgical Research, Anne Arundel Medical Center, Annapolis, USA

**Keywords:** ankle fracture management, closed reduction ankle, complications, postreduction grade, trauma

## Abstract

Background: Ankle fractures are common injuries associated with significant morbidity. A significant proportion of closed ankle fractures are displaced or unstable, requiring reduction. While early near-anatomic reduction is commonly performed, it is not known whether this affects long-term outcomes. This study aims to identify the association of reduction quality with outcomes in patients with displaced, closed ankle fractures. We hypothesize that patients with anatomic reductions will have fewer complications than patients who have suboptimal reductions.

Methods: A retrospective analysis of 167 patients with ankle fractures in the emergency department was conducted. Postreduction X-rays were graded for quality of reduction, and the reducing provider was identified. Patients were grouped by quality of reduction: anatomic and suboptimal. Outcomes, including complications, surgery, and time to surgery, were compared between the groups.

Results: One hundred thirteen (67.7%) patients had an anatomic reduction, and 54 (32.3%) had a suboptimal reduction. Patients with anatomic reduction had higher rates of reduction performed by an orthopedic provider (31.9% vs. 14.8%; p = 0.031). Patients reduced by orthopedics had less talar shift on postreduction X-rays (1.8 ± 1.9 vs. 3.3 ± 4.7; p = 0.005). There were no differences in outcomes between those with anatomic or suboptimal reduction and patients who had a reduction by orthopedics.

Conclusion: Reductions performed by an orthopedic provider were of higher quality; there was no difference in complications between reduction grades and providers. Additional study is needed to determine whether achieving true anatomic reduction is protective against complications and impaired functional outcomes in patients with displaced ankle fractures.

## Introduction

Ankle fractures are one of the most commonly seen fractures in adults, with a recent study determining an incident rate of 4.22/10,000 person-years in the United States [1,2]. Ankle fractures can be a cause of significant long-term morbidity, with up to 70% of patients with ankle osteoarthritis reporting previous ankle trauma [1,3]. While some ankle fractures are closed and nondisplaced, requiring only immobilization, a significant percentage of closed ankle fractures are associated with dislocation, necessitating urgent treatment [3,4]. Unstable and displaced ankle fractures commonly require some sort of surgical intervention, such as open reduction and internal fixation, which can be associated with significant short- and long-term complications such as poor wound healing, skin breakdown, infection, and hardware complications [3,5,6].

Conventional wisdom dictates that displaced ankle fractures should be closed and reduced to near anatomic alignment as soon as possible after injury. While closed reduction is vital when there is neurovascular or skin compromise in ankles that is going to require subsequent surgical intervention, the importance of nonurgent closed reduction is not known [2,7]. There has been some research associating malreduction with subsequent surgical site infection, possibly related to skin swelling and compromise [5,6]. On the other hand, closed reduction, particularly repeated attempts at closed reduction, may cause increased soft tissue swelling and additional osteocartilaginous injury, increasing the risk of complications in the short and long term [2]. There is also little research on whether the specialty of the provider performing the reduction influences the outcomes [8].

This study aims to evaluate patients with ankle fractures undergoing closed reduction in the emergency department (ED) and determine whether the quality of the reduction influences patient outcomes and complications. We hypothesize that patients with anatomic reductions will have improved outcomes compared to patients with suboptimal reductions and that patients reduced by orthopedic providers will be more likely to have anatomic reductions and fewer complications.

## Materials and methods

Study population

This study was deemed exempt by the Institutional Review Board, as it constituted a retrospective review of existing medical records. A retrospective observational study was conducted involving 167 patients who presented to the ED with an ankle fracture and who were subsequently discharged from a single regional health system between January 2018 and November 2023. Patients presenting to the EDs of two acute care hospitals within a single health system were included. Only patients who had postreduction X-rays with anterior-posterior (AP), mortise, and lateral views were graded and included. Only patients who followed up with an orthopedic provider after ED discharge were included. A single physician member of the research team evaluated all X-rays. Patients admitted to the hospital and underwent surgery at the time of initial presentation were excluded from the study.

The study was performed by the Department of Orthopedics at Luminis Health Anne Arundel Medical Center, Annapolis, MD, and Luminis Health Doctors Community Medical Center, Lanham, MD.

Study outcomes

The primary outcomes of interest included time to clinic follow-up, undergoing surgery, the time to surgery, operating room (OR) time, complications (such as deep vein thrombosis/pulmonary embolism (PE), infection, wound complications, nonunion/malunion, and painful hardware/post-traumatic arthritis), and the time to the last orthopedic follow-up.

Independent variables

Independent variables of interest included patient demographics, fracture type, and ED treatment details. Demographics included patient age, sex, and body mass index. Fracture and ED treatment details included fracture type, Weber classification, Maisonneuve fracture, whether Orthopedics was consulted to the ED, failed reduction attempts, and whether an Orthopedic or ED provider performed the final reduction. Orthopedic providers included any board-certified orthopedic surgeon, orthopedic resident physician, or orthopedic-trained advanced practice provider (physician assistant or nurse practitioner). ED providers included any ED physician or advanced practice provider (physician assistant or nurse practitioner).

Statistical analysis

Demographics, details about the fracture type, treatment, and follow-up patterns were extracted from the electronic health record. Patients were divided into two groups by quality of final reduction, anatomic (grade A) and suboptimal (grade B/C), using the following criteria based on a previously published methodology [2]: talar shift of 0-2 mm from the central plumb line of AP or mortise X-ray and the central plumb line bisects talar dome on the lateral X-ray; talar shift of 3-10 mm from the central plumb line of AP or mortise X-ray and central plumb line intersects any portion of talar dome on lateral X-ray; and talar shift of >10 mm from the central plumb line of AP or mortise X-ray and the central plumb line does not intersect any portion of talar dome on the lateral X-ray.

Univariate analysis, including chi-square, Fisher's exact, and independent sample t-tests, was performed to assess differences in demographics, fracture type, ED treatment details, and outcomes between the groups. A secondary analysis was performed to assess the differences in fracture type, ED treatment details, and outcomes between patients who had their final reduction performed by an ED provider and patients who had their final reduction performed by an orthopedic provider. Statistical significance was assessed at p < 0.05. All statistical analyses were performed using R Studio (version 4.2.2 © 2009-2023 RStudio, PBC, Boston, MA).

## Results

Of the 167 patients seen in the ED with an ankle fracture, 113 (67.7%) had an anatomic reduction, and 54 (32.3%) had a suboptimal reduction. There were no demographic differences between these patients (Table 1).

**Table 1:** Demographics by quality of reduction All data are presented as n (%) or mean ± SD t-score indicates two-sided t-test; X^2^ indicates chi-square test BMI: body mass index; SD: standard deviation

Demographics	Anatomic (n = 113)	Suboptimal (n = 54)	Test statistic	p value
Age (years)	49.8 ± 17.2	50.4 ± 17.3	t-score = -0.19	0.853
Sex				
Female	75 (66.4)	39 (72.2)	X^2^ = 0.34	0.561
Male	38 (33.6)	15 (27.8)		
BMI (kg/m^2^)	29.7 ± 7.5	31 ± 7.1	t-score = -1.02	0.309

The distribution of single malleolar, bimalleolar, and trimalleolar fractures, Weber classifications, Maisonneuve fractures, and failed reduction attempts was similar between groups. Orthopedics was consulted similarly in both groups, but patients with an anatomic reduction had higher rates of final reduction performed by an orthopedic provider (31.9% vs. 14.8%; p = 0.031) compared to those with suboptimal reductions. After reduction, patients with a suboptimal reduction had greater talar shift from the central plumb line (5.9 ± 5.8 vs. 1.4 ± 1.9; p < 0.001), had higher rates of the central plumb line intersecting of the talar dome (33.3% vs. 0%; p < 0.001), and lower rates of the central plumb bisecting the talar dome (66.7% vs. 100%; p < 0.001) compared to anatomic reductions (Table 2).

**Table 2:** Fracture and ED treatment details by quality of reduction All data are presented as n (%) or mean ± SD ED: emergency department; AP: anteroposterior; SD: standard deviation t-score indicates two-sided t-test; X^2^ indicates chi-square test ^*^Fisher’s exact test ^†^p value <0.05

Fracture and ED treatment details	Anatomic (n = 113)	Suboptimal (n = 54)	Test statistic	p value
Fracture type				
Single malleolar	14 (12.4)	2 (3.7)	X^2^ = 3.21	0.201
Bimalleolar	33 (29.2)	18 (33.3)		
Trimalleolar	66 (58.4)	34 (63.0)		
Maisonneuve fracture	5 (4.4)	1 (1.9)	-	0.665^*^
Weber class				
A	7 (6.2)	2 (3.7)	-	0.905^*^
B	82 (72.6)	41 (75.9)		
C	19 (16.8)	10 (18.5)		
Orthopedics consulted	53 (46.9)	16 (29.6)	X^2^ = 3.81	0.051
Failed reduction attempts				
0	90 (79.6)	41 (75.9)	-	0.709^*^
1	21 (18.6)	11 (20.4)		
2	2 (1.8)	2 (3.7)		
Final reduction by orthopedics	36 (31.9)	8 (14.8)	X^2^ = 4.63	0.031^†^
AP/mortise talar shift (mm)	1.4 ± 1.9	5.9 ± 5.8	t-score = -5.57	<0.001^†^
Lateral bisects talar dome	113 (100)	36 (66.7)	X^2^ = 38.82	<0.001^†^
Lateral intersects talar dome	0 (0)	18 (33.3)	-	<0.001^*†^
Lateral does not intersect talar dome	0 (0)	1 (1.9)	-	0.323^*^

There were no differences in outcomes between anatomic and suboptimal reductions. Patients with suboptimal reductions had significantly longer follow-ups than those with anatomic reductions (226.9 ± 265.8 vs. 147.6 ± 139.7; p = 0.043) (Table 3).

**Table 3:** Outcomes and follow-up patterns by quality of reduction All data are presented as n (%) or mean ± SD OR: operating room; DVT: deep vein thrombosis; PE: pulmonary embolism; SD: standard deviation t-score indicates two-sided t-test; X^2^ indicates chi-square test ^*^Fisher’s exact test ^†^p value <0.05

Outcome	Anatomic (n = 113)	Suboptimal (n = 54)	Test statistic	p value
Time to clinic follow-up	5.5 ± 9.0	4.1 ± 4.2	t-score = 1.34	0.134
Clinic follow-up >7 days	19 (16.8)	10 (18.5)	X^2^ = 0.003	0.957
Underwent surgery	94 (83.2)	50 (92.6)	X^2^ = 1.99	0.159
Time to surgery	10.3 ± 6.9	10.6 ± 9.6	t-score = -0.17	0.965
Minutes in OR	108.2 ± 47.9	108.6 ± 40.4	t-score = -0.050	0.960
Any complication	24 (21.1)	12 (22.2)	X^2^ = 2.79 e^-30^	1
DVT/PE	1 (0.9)	0 (0)	-	1^*^
Infection	2 (1.8)	0 (0)	-	1^*^
Wound complication	8 (7.1)	6 (11.1)	-	0.384^*^
Nonunion/malunion	5 (4.4)	1 (1.9)	-	0.665^*^
Painful hardware/posttraumatic arthritis	8 (7)	5 (9.3)	-	0.758^*^
Time to last ortho follow-up	147.6 ± 139.7	226.9 ± 265.8	t-score = -2.06	0.043^†^

When comparing those whose final reduction was performed by an ED provider and those whose final reduction was performed by an orthopedic provider, there were no differences in fracture type, Weber classification, Maisonneuve fractures, failed reduction attempts, or central plumb line bisection/intersection of the talar dome. However, on average, patients who were reduced by orthopedics had less talar shift in the postreduction X-rays (1.8 ± 1.9 vs. 3.3 ± 4.7; p = 0.005) compared to those who had their reduction performed by an ED provider. Additionally, there were more anatomic reductions when the final reduction was performed by an orthopedic provider (81.8% vs. 62.6%; p = 0.026) (Table 4).

**Table 4:** Fracture and ED treatment details by final reduction provider All data are presented as n (%) or mean ± SD ED: emergency department; AP: anteroposterior t-score indicates two-sided t-test; X^2^ indicates chi-square test ^*^Fisher’s exact test ^†^p value <0.05

Fracture and ED treatment details	Final reduction by ED provider (n = 123)	Final reduction by orthopedic provider (n = 44)	Test statistic	p value
Fracture type				
Single malleolar	8 (6.5)	8 (18.2)	X^2^ = 5.31	0.070
Bimalleolar	40 (32.5)	11 (25)		
Trimalleolar	75 (61)	25 (56.8)		
Maisonneuve fracture	4 (3.3)	2 (4.5)	-	0.654^*^
Weber class				
A	5 (4.1)	4 (9.1)	-	0.400^*^
B	93 (75.6)	30 (68.2)	-	
C	21 (17.1)	8 (18.2)	-	
Failed reduction attempts				
0	97 (78.9)	34 (77.3)	-	0.551
1	24 (19.5)	8 (18.2)	-	
2	2 (1.6)	2 (4.5)	-	
Quality of reduction				
Anatomic	77 (62.6)	36 (81.8)	X^2^ = 4.63	0.031^†^
Suboptimal	46 (37.4)	8 (17.8)		
AP/mortise talar shift (mm)	3.3 ± 4.7	1.8 ± 1.9	t-score = 2.82	0.005^†^
Lateral bisects talar dome	109 (88.6)	40 (90.9)	-	0.784^*^
Lateral intersects talar dome	14 (11.4)	4 (9.1)	-	0.784^*^
Lateral does not intersect talar dome	0 (0)	1 (2.3)	-	0.264^*^

There were no differences in outcomes between reductions performed by an ED provider and reduction performed by an orthopedic provider (Table 5).

**Table 5:** Outcomes and follow-up patterns by final reduction provider All data are presented as n (%) or mean ± SD OR: operating room; DVT: deep vein thrombosis; PE: pulmonary embolism; SD: standard deviation t-score indicates two-sided t-test; X^2^ indicates chi-square test ^*^Fisher’s exact test

Outcome	Final reduction by ED provider (n = 123)	Final reduction by orthopedic provider (n = 44)	Test statistic	p value
Time to clinic follow-up	5.3 ± 8.8	4.3 ± 3.8	t-score = 1.08	0.280
Clinic follow-up >7 days	19 (15.4)	10 (22.7)	X^2^ = 0.74	0.389
Underwent surgery	108 (87.8)	36 (81.8)	X^2^ = 0.54	0.376
Time to surgery	10.1 ± 8.4	11.3 ± 6.4	t-score= -0.90	0.373
Minutes in OR	106.5 ± 47.8	114.1 ± 36.5	t-score= -0.90	0.372
Any complication	29 (23.6)	7 (15.6)	X^2^ = 0.72	0.363
DVT/PE	0 (0)	1 (2.2)	-	0.268^*^
Infection	2 (1.6)	0 (0)	-	1^*^
Wound complication	12 (9.8)	2 (4.5)	-	0.359^*^
Nonunion/malunion	4 (3.3)	2 (4.5)	-	0.659^*^
Painful hardware/posttraumatic arthritis	11 (8.9)	2 (4.5)	-	0.517^*^
Time to last ortho follow-up	167.5 ± 172.5	189.1 ± 241.9	t-score = -0.54	0.588

## Discussion

Patients with ankle fractures undergoing initial reduction in the ED had similar outcomes regardless of the quality of the final reduction or the provider performing the reduction. Those with a reduction performed by an orthopedic provider were likelier to have a high-quality anatomic reduction and less talar shift. However, this did not ultimately affect surgery rates or complication rates in these patients. In the current study, only 68% of patients were discharged from the ED with anatomic reductions. This finding is similar to those of Chien et al., who observed a rate of 61.5% grade A reductions using the same grading methodology in their study of 161 ankle fractures [2]. In a historical study of 78 patients with severe malleolar fractures treated with closed reduction, Federici et al. reported that anatomical reduction was achieved in only 32.4% of patients [9]. Collectively, these findings suggest that the quality of closed reductions for patients suffering ankle fractures is highly variable, with a significant number of patients discharged with some level of malalignment. While it is clear that opportunities for improving the quality of closed reductions performed in the ED exist, the impact of initial reduction quality on patient outcomes remains a topic of debate.

Achieving anatomic reduction of ankle fractures has been shown to affect functional outcomes, quality of life, mobility, and patient satisfaction [2,10,11]. In a study of 237 patients with ankle fractures of varying severity, the patient perception and satisfaction with the ultimate outcome were correlated with the radiographic result but not with the type of treatment (operative or nonoperative), type of injury, or patient age [12]. In a study involving 620 adults over the age of 60, patients had equivalent functional outcomes at six months and three years, regardless of whether they underwent closed reduction and casting or open reduction, as long as anatomic reduction was maintained [10,13]. In this study, there was no difference in rates of nonunion/malunion, hardware complications, or posttraumatic arthritis when comparing anatomic reductions with suboptimal reductions. However, we did not examine functional or patient-reported outcomes. We did, however, note a difference in follow-up time when comparing reduction quality; patients with a suboptimal reduction required more than two months of additional orthopedic follow-up on average. This may indicate that patients with a suboptimal initial reduction, in fact, did have a decrease in functional outcomes or required a longer course of treatment to get back to their desired level of activity when compared to patients who had an anatomic initial reduction.

Unreduced or poorly reduced ankle fractures place increased stress on the skin and soft tissue; numerous studies have shown that adequate closed reduction reduces the risk of complications, particularly skin and soft tissue complications [6,14-16]. A study by Ovaska et al. involving 1,923 ankle fractures revealed a 3.4-fold increase in the risk of surgical site infection for malreduced ankles [6]. Inadequate reduction can also exacerbate soft tissue swelling and delay definitive operative intervention, which is a risk factor for postoperative infectious complications as well [5,17]. On the other hand, repeated manipulation of the joint to obtain an ideal reduction can exacerbate soft tissue swelling and osteocartilaginous injury; Chien et al. found that there was no difference in wound complications of patients with more poorly graded reductions, although patients with the least ideal reductions did proceed to surgery sooner [2]. A study by Novack et al. found that when surgical intervention is planned, there was no difference in patient outcomes at one year, whether or not an adequate reduction was achieved initially [7]. Our results more closely align with those of Chien et al. and Novack et al., as we showed no difference in infection or wound complications when comparing the anatomic reductions with the suboptimal reductions. However, we also noted no difference in time to surgery between the groups. Nearly 80% of the patients in this study only required a single attempt at reduction as well, which may have limited the risk of additional injury from repeated reduction attempts.

Depending on the setting, various specialists can perform ankle fracture reductions. In the case of neurovascular compromise, reductions are often performed in the prehospital setting [17]. In the ED, reductions can be performed by emergency providers, orthopedic providers, or podiatric providers. Little research has been performed comparing the effectiveness of ankle reductions performed by each group. Levi et al. found no difference in whether the initial ankle fracture reduction was successful when performed by an emergency medicine physician, podiatrist, or orthopedic surgeon [8]. When examining surgical outcomes as opposed to initial closed reduction, Tenenbaum et al. found that ankle fracture surgery performed by orthopedic surgical residents achieved equivalent results to surgery performed by fellowship-trained foot and ankle or trauma surgeons [18]. In this study, closed reductions performed by orthopedic providers were more likely to result in a higher quality reduction with less talar shift on the radiograph. However, this ultimately led to no significant differences in outcomes. These findings raise an interesting question regarding appropriately allocating limited provider resources to ankle fracture patients. While our data suggest that a lower threshold for consulting orthopedic specialists to perform difficult reductions may be warranted, it also indicates that the initial reduction quality does not significantly affect outcomes. However, until a consensus regarding the relationship between reduction quality and outcomes is reached through further studies, we suggest early involvement of the orthopedic service in managing complex ankle fractures.

The results of this study need to be considered in light of its limitations. It is a retrospective study from a single institution, and as such, the results may not be comparable to a wider patient population. Further, patients without postreduction radiographs or orthopedic follow-up were excluded, which may bias the results. In addition, the follow-up time was relatively short and did not include patient-reported or functional outcomes, which would provide valuable information regarding the importance of initial reduction quality. Finally, the sample size is relatively small, and the complication rates were low, which may leave the study underpowered to detect significant differences. Despite these limitations, we feel this study is a valuable contribution to the literature as it is one of the few studies assessing the quality of ankle fracture reductions and the relationship between reduction quality and the risk of postoperative complications in patients with displaced, closed ankle fractures.

## Conclusions

For patients seen in the ED undergoing closed reduction of displaced ankle fractures, the overall quality of reduction was moderate and most patients only needed a single reduction attempt. Reductions performed by an orthopedic provider were more likely to be higher quality with less talar shift, although there was no difference in complications when comparing reduction quality or reduction provider. Additional study is needed to determine whether achievement of true anatomic reduction is protective against complications and impaired functional outcomes in patients with displaced ankle fractures.
